# New Attractant Lures for Sampling *Conistra vaccinii* L. Populations: Bisexual Lures and a Sex Attractant (Lepidoptera: Noctuidae)

**DOI:** 10.3390/insects16020177

**Published:** 2025-02-07

**Authors:** Szabolcs Szanyi, Zoltán Varga, Antal Nagy, Gábor Szőcs, Júlia Katalin Jósvai, Miklós Tóth

**Affiliations:** 1 Faculty of the Agricultural and Food Sciences and Environmental Management, Institute of Plant Protection, University of Debrecen, H-4002 Debrecen, Hungary; nagyanti@agr.unideb.hu; 2 Department of Evolutionary Zoology and Human Biology, University of Debrecen, Egyetem tér 1, H-4010 Debrecen, Hungary; varga.zoltan@science.unideb.hu; 3 Plant Protection Institute, CAR, HUN-REN, H-1525 Budapest, Hungary; szocs.gabor@atk.hun-ren.hu (G.S.); josvai.julia@atk.hun-ren.hu (J.K.J.); toth.miklos@atk.hun-ren.hu (M.T.)

**Keywords:** synthetic lures, semi-synthetic lure, chemical ecology, feeding attractant, trapping

## Abstract

The chemical ecology and host plant preferences of the different Conistra species (Lepidoptera: Noctuidae, Xylenini) are mostly unknown, even though they are essential components of the herbivorous assemblages of temperate zone forests. During field studies, (Z)-7-tetradecenyl acetate (Z7-14Ac) was identified as a sex attractant of *Conistra vaccinii*. Phenylacetaldehyde-based synthetic and iso-amyl alcohol-based semi-synthetic lures also attracted *C. vaccinii*, *C. rubiginea*, and *C. rubiginosa*, and in the case of *C. vaccinii*, they performed better than the newly identified sex attractant. The different efficiencies of the two bisexual lures probably reflected the different feeding source preferences of the species caught. The tested lures attracted both sexes and, thus, we can estimate the actual sex ratio. These traps can be used especially in applications where female samples are needed, i.e., surveys on the reproduction biology of moths (e.g., polyandry), or in surveys of population dynamics.

## Simple Summary

Conistra species belonging to the owl moths (Noctuidae) are important members of the herbivorous insect assemblages of temperate forests. Here, we described (Z)-7-tetradecenyl acetate (Z7-14Ac) as a sex attractant of *C. vaccinii*. Additionally, the attractivity of phenylacetaldehyde-based synthetic and isoamyl alcohol-based semi-synthetic lures to different Conistra species was also proved, showing the different food source preferences of the species attracted. Using the new sex attractant, we can catch only males, while the other two lures attract both sexes. Depending on the goal of further studies, one or other of these new lures can serve as an appropriate tool for study on these widely distributed members of arboreal fauna.

## 1. Introduction

Conistra is a highly diverse Eurasiatic genus of the noctuid tribe Xylenini, consisting of more than forty species, subdivided into four subgenera and several species groups [1,2]. Most of these species occur in the warm-temperate belts of Europe and Southeast Asia, inhabiting forested habitats. The larvae are mostly polyphagous in early stages with some preferences for woody or shrubby plants, but in later stages, they often feed on herbaceous plants. In Europe, Malus, Prunus, Ribes, Rubus, Betula, Quercus, Salix, etc. species, and *Vaccinium myrtillus* are mentioned [3], while in East Asia different Betulaceae, Fagaceae, Malvaceae, Rosaceae, and Sapindaceae species are also considered as food plants [4]. Conistra species are important and sometimes also abundant components of the moth assemblages of rich deciduous forests; however, they were never mentioned as pest species. The adults are regularly more attracted by different traditional fermenting sugar baits than by light traps [1]. However, this mostly anecdotal information is not supported by quantitative data, which could show differences in the preferences of different species.

In recent decades, pheromone structures of many noctuids have been characterized, and also, sex attractants of several species have been discovered by screening of known synthetic pheromone components. In the latter cases, however, no information is available on whether females of the given species indeed produced the compound in question, which attracted males in the field [5]. Traps baited with these synthetic compounds are suitable for field sampling of males of numerous moths of both pest and non-pest species.

More recently, bisexual synthetic lures have been developed, which would have the advantage of attracting both sexes of the target species. Having analyzed volatiles emanating from fermenting liquids (frequent feeding sources of many noctuids), iso-amyl alcohol and acetic acid were identified [6]. These components were merged in a synthetic lure, which proved to be attractive to many noctuids in North America [7] and also in Europe [8]. In more recent studies in Hungary, with the addition of the natural ingredients of red wine extract or red wine to iso-amyl alcohol plus acetic acid containing lures, a more powerful semisynthetic bisexual lure (SBL) was developed [9]. This attracted a great number of noctuid taxa in Hungary and Transcarpathia (West Ukraine), especially species of the subfamilies Acronictinae, Amphipyrinae, and Noctuinae, including Hadenini, Noctuini, and Xylenini [10,11,12].

On the other hand, it has long been known that the common floral compound phenylacetaldehyde also attracts numerous noctuid moth species [13,14]. The addition of some other synthetic floral compounds resulted in optimized bisexual attractants for several noctuids [15,16,17,18]. Based on these studies, a four-component blend of floral compounds (FLO) was developed, consisting of phenylacetaldehyde, (*E*)-anethol, benzyl acetate, and eugenol, which attracted significant numbers of mainly Plusiinae and Melicleptriinae species in the field [10,11,12].

The objective of the present study was to develop a sex attractant lure and to compare it with bisexual lures, which would make the successful field sampling of *Conistra vaccinii* populations possible but also of some congeneric, less common species. *C. vaccinii* was selected as the focal target species of this survey since this species is one of the most common components of the autumnal and early spring moth assemblage of deciduous forest habitats. Its successful sampling is a pre-requisite to test some hypotheses concerning the feeding and reproductive behavior of this and also related species, e.g., differences in pre- vs post-hibernation behavior, period of fertilization, the level of polyandry and its relation to the well-known high variability of this species, etc.

## 2. Materials and Methods

### 2.1. Field Tests

Tests were conducted using well-tried methods in similar trapping experiments [19] at four sites in Hungary. Traps were arranged in a large circle, in a random order, separated by 8–10 m. Traps were sampled preferably twice weekly, depending on weathering conditions, when captured insects were removed and recorded. At the same time, traps were moved one position forward in the circle to minimize the effect of the sampling location. The captured specimens were identified based on standard works on European [1] and Hungarian Noctuidae [20]. In dubious cases (damaged specimens), the genitalia were tested.

Field trapping data did not fulfil the statistical requirements for a parametric analysis (by Q–Q plots and Levene’s test), therefore, data were analyzed by the non-parametric Kruskal–Wallis test. If the Kruskal–Wallis test yielded significance (P = 5%), then treatments were compared pairwise by a Mann–Whitney U test. All statistical procedures were conducted using the software packages StatView^®^ v4.01 and SuperANOVA^®^ v1.11 (Abacus Concepts, Berkeley, CA, USA).

### 2.2. Traps

In the tests, CSALOMON^®^ VARL funnel traps (Plant Protection Institute, HUN-REN CAR, Budapest, Hungary) were used. These traps have routinely been used for trapping several noctuids [8], including *Conistra* spp. [21]; photos of the trap can be viewed at www.csalomontraps.com (accessed on 11 November 2024). For killing captured insects, a small piece (1 × 1 cm) of a household anti-moth insecticide strip (Chemotox^®^ SaraLee, Temana Intl. Ltd., Slouth, UK; active ingredient 15% dichlorvos) was placed into the catch container of traps.

### 2.3. Baits

The tested semisynthetic bisexual lure (SBL) was the same type as described earlier [9,22], with the active ingredients of iso-amyl alcohol, acetic acid, and red wine (1:1:1).

The synthetic FLO lure contained phenylacetaldehyde, (*E*)-anethol, benzyl acetate, and eugenol (1:1:1:1) [12]. For details of the preparation of these two lures, please refer to [9,12,21,22].

Sex attractant (throughout the paper, we use the commonly accepted nomenclature for sex attractants vs. pheromones established in Arn et al., 1986 [23]) candidate lures were prepared by adding the necessary amounts of (Z)-7-tetradecenyl acetate (Z7-14Ac), (Z)-7-tetradecenol (Z7-14OH), and (Z)-7-tetradecenal (Z7-14Ald) on 1 cm pieces of rubber tubing (Taurus, Budapest, Hungary; No. MSZ 9691/6; extracted 3 times in boiling ethanol for 10 min, and then also 3 times in methylene chloride overnight) in hexane solutions. After having allowed the solvent to evaporate, dispensers were wrapped singly in pieces of aluminium foil and were stored below −10 °C until use. The synthetic samples were generous gifts from Marie Bengtsson (Alnarp, Sweden) (Z7-14Ald) and the late Simon Voerman (Wageningen, The Netherlands) (Z7-14Ac and Z7-14OH), and were >98% purified by gas chromatography.

### 2.4. Experimental Details

**Figure 1:**
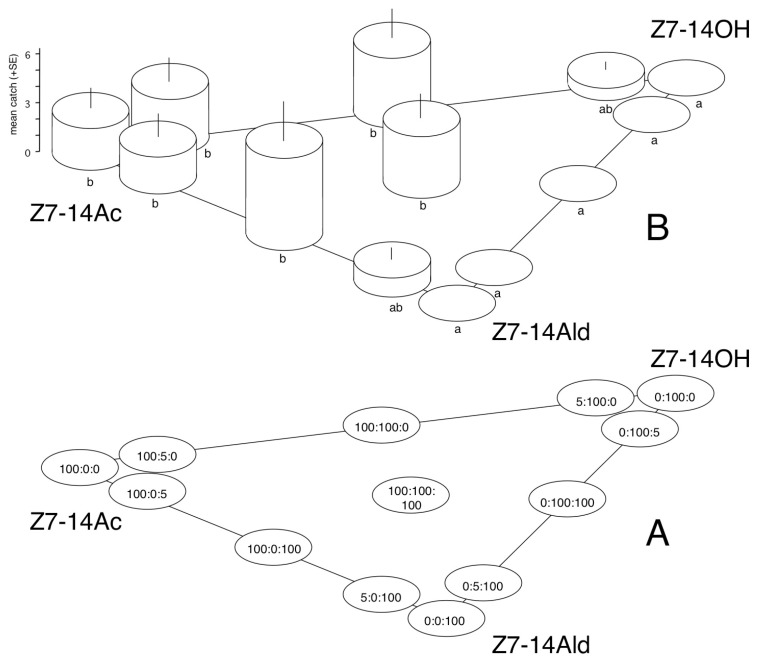
Catches of *Conistra vaccinii* in traps baited with (Z)-7-tetradecenyl acetate (Z7-14Ac), (Z)-7-tetradecenol (Z7-14OH), (Z)-7-tetradecenal (Z7-14Ald), and their binary and ternary mixtures. (**A**): composition of lures (µg); (**B**): distribution of catches between the different lures. Columns with the same letter within the diagram (**B**) are not significantly different as determined by a Kruskal–Wallis test and followed by pairwise comparisons by a Mann–Whitney U test (P = 5%).

Experiment 1. As part of a widespread screening of known noctuid sex pheromone components for defining sex attractants, lures containing synthetic Z7-14Ac preliminarily showed some captures; consequently, Z7-14Ac, Z7-14OH, Z7-14Ald, and their binary and ternary mixtures were tested (for variations, refer to Figure 1A). From each treatment, 2 traps were set out, giving a total number of 28 traps. The test was run from 8 March to 16 April 1992. The test was conducted at the Julianna major Experimental Station of the Plant Protection Institute of the Centre for Agricultural Research, Eötvös Loránd Research Network (PPI CAR ELKH, Budapest, Hungary) in a mixed sessile-turkey oak forest.

Experiment 2. Treatments tested were the SBL lure, the FLO lure, and the synthetic sex attractant of *C. vaccinii* (100 µg Z7-14Ac on rubber dispenser, characterized in Exp. 1), and unbaited controls. From each treatment, 4 traps were set out, giving a total number of 16 traps. The test was run from 2 November 2018 to 11 April 2019. Traps were not visited between 4 December 2018 and February 28, 2019; before and after these dates, regular inspections were performed and continued to the end of the test period. Lure dispensers were replaced by new ones on 28 February 2019. The test was conducted at the same site as Exp. 1.

Experiment 3. Treatments tested included the SBL lure, the FLO lure, and unbaited traps. From each treatment, 4 traps were set out, giving a total number of 12 traps. The tests were run in parallel at 3 sites.

Exp. 3A: Jánkmajtis, Szabolcs-Szatmár-Bereg county, 1 March–18 April 2020, in the margin of a lowland oak gallery forest.

Exp. 3B: Nyírbátor, Szabolcs-Szatmár-Bereg county, 2 March–20 April 2020, in the margin of a mixed willow-poplar forest plantation.

Exp. 3C: Julianna major, Budapest, 27 February–15 April 2020, mixed sessile-turkey oak forest.

## 3. Results

In the catch, *Conistra* and *Orthosia* spp. were the most abundant. In this present paper, we discuss in detail only results on the *Conistra* spp. (*C. vaccinii* Linn., *C. erythrocephala* Den. et Schiff., *C. rubiginea* Den. et Schiff., and *C. rubiginosa* Scop.); capture results on *Orthosia* spp. and other noctuids caught in lower numbers have already been published [12,19,22].

*C. vaccini*—In Exp. 1, sizeable numbers of *C. vaccinii* were caught exclusively in traps with baits containing 100 µg of Z7-14Ac (no matter whether Z7-14OH or Z7-14Ald were also present in binary or ternary combinations); catches were significantly more than treatments with zero catch (Figure 1). Low catches (not significantly different from zero catch) were recorded in two treatments, containing 5 µg of Z7-14Ac (with 100 µg of Z7-14OH or Z7-14Ald as second components). All captured specimens were males. No noctuids belonging to other species were captured.

**Figure 2:**
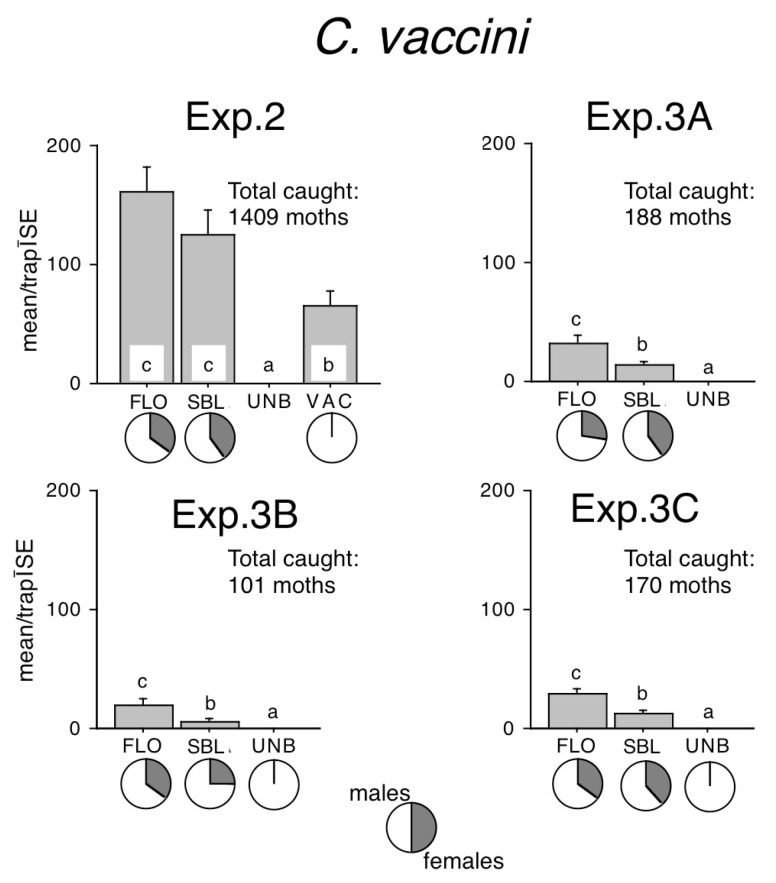
Catches of *Conistra vaccinii* in traps baited with bisexual lures FLO and SBL, or with the synthetic sex attractant (VAC), and in unbaited traps (UNB). Columns with the same letter within one diagram are not significantly different as determined by a Kruskal–Wallis test and followed by pairwise comparisons by a Mann–Whitney U test (P = 5%). Pie charts under the columns show the sex ratio in the catch.

In Exp. 2, no specimens were captured until the last inspection before winter (6 December 2018), while already good catches were recorded on the first inspection in early spring on 28 February 2019 and onwards. Sizeable numbers were also caught at all sites of Exp. 3 (Figure 2).

Traps baited with the sex attractant caught many times more moths than unbaited traps (Figure 2). However, significantly more moths were recorded in both FLO and SBL, which did not differ from each other, although numerically FLO caught the most moths (Figure 2, Exp. 2). In each site of Exp. 3, FLO caught significantly more than SBL, and both lures caught better than unbaited traps, which caught zero or occasionally a single stray specimen (Figure 2).

The traps baited with the sex attractant caught only males, while those with semi-synthetic and synthetic bisexual lures caught a sizeable proportion of females in all experiments (Figure 2). Female ratios in FLO–baited traps ranged from 28% to 36%, and in SBL–baited traps from 26% to 40%, and female ratios within one site were remarkably similar in FLO or SBL (Figure 2).

**Figure 3:**
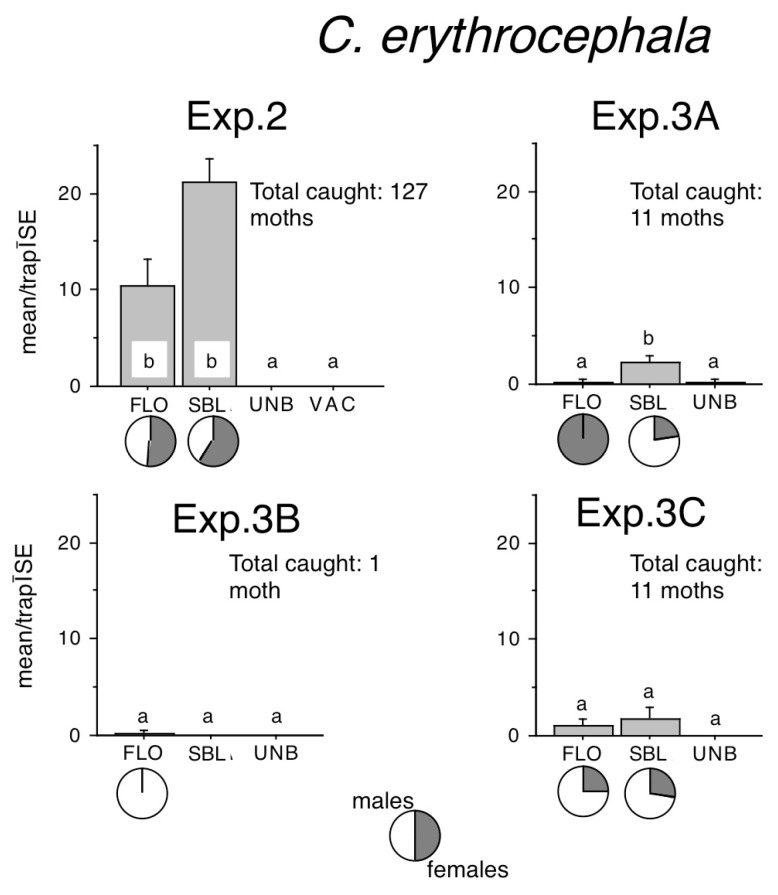
Catches of *Conistra erythrocephala* in traps baited with bisexual lures FLO and SBL, or with the synthetic sex attractant of *Conistra vaccinii* (VAC), and in unbaited traps (UNB). Columns with the same letter within one diagram are not significantly different as determined by a Kruskal–Wallis test and followed by pairwise comparisons by a Mann–Whitney U test (P = 5%). Pie charts under the columns show the sex ratio in the catch.

Sizeable *C. erythrocephala* captures were recorded only in Exp. 2. (Figure 3). FLO or SBL–baited traps caught significantly more than zero catch in unbaited traps. Although numerically SBL caught twice as much as FLO, the difference was not significant. Lower catches in Exp. 3A and 3C showed the same trend (Figure 3).

In Exp. 2, female ratios in FLO were 53%, while in SBL, they were 59% (Figure 3). Catches in Exp. 3A and 3C were too low to allow for a meaningful discussion of female ratios.

**Figure 4:**
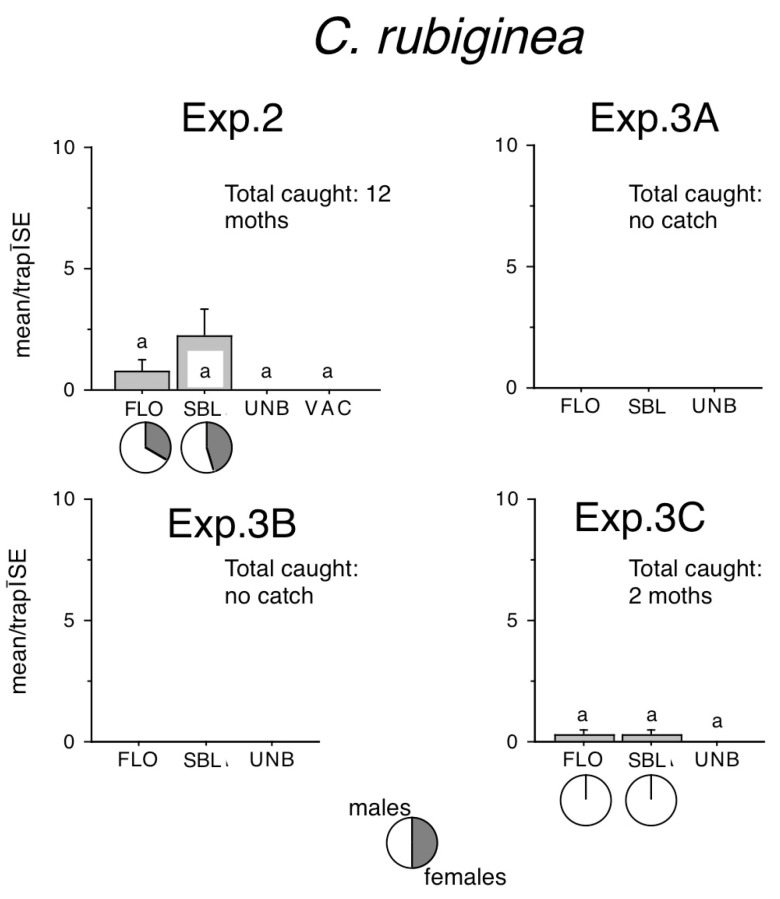
Catches of *Conistra rubiginea* in traps baited with bisexual lures FLO and SBL, or with the synthetic sex attractant of *Conistra vaccinii* (VAC), and in unbaited traps. Columns with the same letter within one diagram are not significantly different as determined by a Kruskal–Wallis test and followed by pairwise comparisons by a Mann–Whitney U test (P = 5%). Pie charts under the columns show the sex ratio in the catch.

A total of 12 *C. rubiginea* moths were recorded in Exp. 2, and 2 moths in Exp. 3C (Figure 4) in FLO and SBL–baited traps. No significant difference was found between zero catches of unbaited traps and any other treatments.

**Figure 5:**
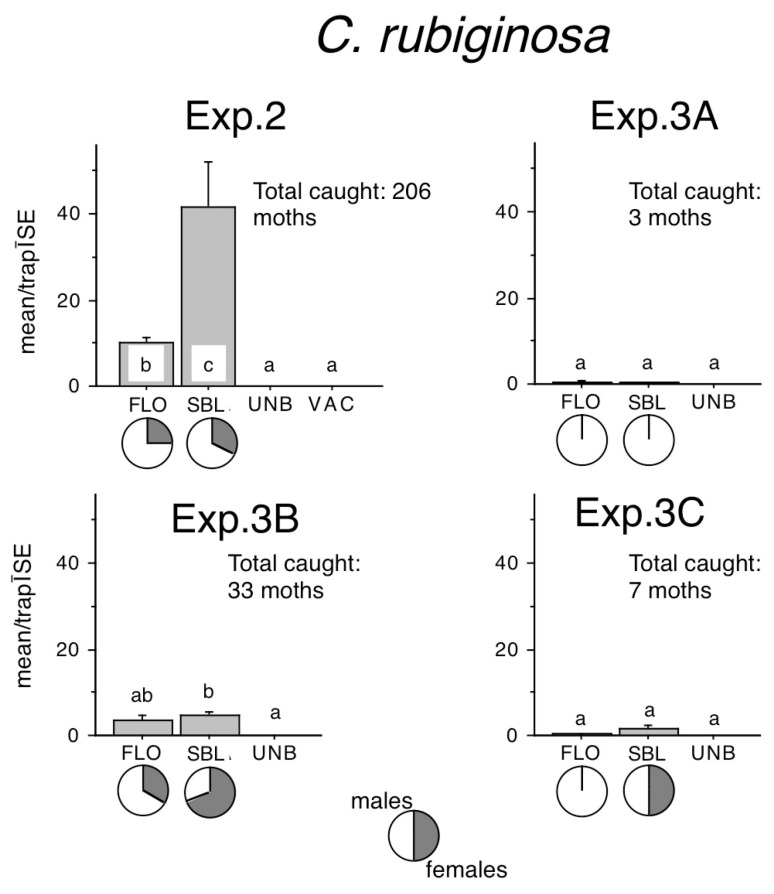
Catches of *Conistra rubiginosa* in traps baited with bisexual lures FLO and SBL, or with the synthetic sex attractant of *Conistra vaccinii* (VAC), and in unbaited traps (UNB). Columns with the same letter within one diagram are not significantly different as determined by a Kruskal–Wallis test and followed by pairwise comparisons by a Mann–Whitney U test (P = 5%). Pie charts under the columns show the sex ratio in the catch.

Sizeable *C. rubiginosa* captures were recorded only in Exp. 2. and Exp. 3B (Figure 5). FLO–baited traps caught significantly more than unbaited traps only in Exp. 2, while SBL caught more than unbaited traps in both experiments. SBL–baited traps also caught better than FLO–baited traps in Exp. 2. Lower catches in Exp. 3A and 3C were too low to allow for meaningful statistical analysis (Figure 5).

Female ratios in FLO–baited traps ranged from 25% to 33%, and in SBL–baited traps from 33% to 68%, and female ratios in Exp. 2 were remarkably similar in FLO or SBL (Figure 5).

## 4. Discussion

Results in Exp. 1 clearly suggest Z7-14Ac to be a sex attractant of *C. vaccinii*. The presence of the corresponding alcohol or aldehyde did not show any influence on the activity of the acetate. The present results confirmed an earlier report by the late Ernst Priesner (pers. comm. [23]) on Z7-14Ac being attractive (with the classification of A = sex attractant) to *C. vaccinii*, but no other details are available. Since in the Conistra genus no female-produced pheromones have been characterized yet [5], further studies are needed to confirm whether *C. vaccinii* females also produce the same compound in the natural sex pheromone emitted.

The compound Z7-14Ac occurs relatively frequently among the pheromone components of noctuid moths. [5] lists several dozen noctuid spp. where Z7-14Ac plays some role in sexual communication. Within the Conistra genus, none of the five species for which sex attractant composition is given so far [5] have been described to use Z7-14Ac.

Catches by Z7-14Ac appeared to be highly specific in the present study, so we concluded that it could be recommended for use as a synthetic bait in traps for sampling *C. vaccinii* males.

In the present study, *C. vaccinii* responded well to both types of bisexual lures, although catches of floral FLO were generally higher (in three out of four cases significantly so) than fermenting liquid-originated SBL. This suggests that floral feeding sources might be preferred and more important for this species than fermenting liquid feeding sources. These data and also the high catches from February suggest that this generally abundant species may have a considerable role in the early spring pollinator assemblages.

In early spring, adults of *C. vaccinii* were frequently seen feeding on amenta of willow species (*Salix* spp.) [24]. When analyzing effluents of flowering and non-flowering *Salix fragilis* and *S. rubens* twigs (Salicaceae), the presence of none of the floral components of the FLO lure was reported [25]. Conversely, *Conistra* spp. were attracted in the autumn by fermenting fruits [26], consisting of a more balanced complement of nutrients, e.g., not only carbohydrates but also amino acids. In the future, the efficacy of the FLO lure could further be increased by applying compounds characterized by [25], e.g., 1,4-dimethoxybenzene and (E)-β-ocimene, which were reported by them as the most abundant components in flowering willow effluents.

On the other hand, the other three *Conistra* spp. captured in the present study appeared to respond to the bisexual lures in an opposite way, preferring the fermenting SBL lure over FLO. Further studies can clarify the feeding source preferences of *Conistra* spp.

In the present study, both bisexual lures caught more significant *C. vaccinii* than the traps with the sex attractant Z7-14Ac. The good performance of the SBL lure vs. Z7-14Ac confirms the trends observed in an earlier preliminary comparison of the SBL lure and Z7-14Ac at very low population densities, in which SBL caught comparable amounts to Z7-14Ac; however, the differences were not significant [21].

The outstandingly good performance of the FLO lure vs. the sex attractant of *C. vaccinii* seems to go against the general trend in noctuids. In most cases, sex pheromone or attractant-baited traps catch more of a given moth species than bisexual lures [17,18,21,27]. An explanation for the lower activity of Z7-14Ac in *C. vaccinii* may be that either the sex attractant composition of *C. vaccinii* is not yet optimized, or the sex-related response of this species is not very strong. We could only resolve this question by the exact identification of the chemical composition of the female pheromones of the Conistra species. Whenever the female-produced pheromone composition of *C. vaccinii* is identified, new and better compositions could be prepared and experimentally tested to uncover individual variations in the responses of males [28,29].

A comparison of sex attractant vs. bisexual lures was not possible in the other three *Conistra* spp. caught. Although a sex attractant consisting of (Z)-11-hexadecenyl acetate and (Z)-11-hexadecenal has been reported for *C. rubiginea* [30], in numerous field tests of our own of various mixtures of these two compounds, we never observed catches of more than a handful of specimens from this species, so we supposed that the sex attractant has not been sufficiently optimized for Conistra species and, thus, was not included in the present experiments.

The bisexual lures caught both sexes of all *Conistra* spp., and a sizeable percentage of the catch was females. This is probably due to the fact that females need energy and vitamins to be able to produce viable eggs, so it is not surprising that a feeding stimulus is important to them. It is known that *Conistra* spp. and other noctuids with a similar life history are “income breeders” [26] since their reproductive success strongly depends on the nutrient intake of the adults. This characteristic of the bisexual lures can be very effectively exploited in applications where the capture of females is needed, i.e., practically all surveys on the reproduction biology of moths (e.g., polyandry), or in population dynamics in which the sex ratio may be a significant factor. Wherever an experiment resulted in sizeable numbers of moths in the present study, female ratios in the two types of bisexual lures were strikingly similar within a site in all *Conistra* spp. This suggests that the relative responsiveness of the sexes was similar to both the floral and fermenting types of lures, and sex ratios in the catch would approximately reflect the actual sex ratio of the given population. However, to experimentally prove this, independent measurements of the natural sex ratios should be conducted in the future.

In conclusion, in the present study, FLO, which is a new, more efficient bisexual lure of a floral type, was described for sampling both sexes of *C. vaccinii*, which could advantageously be used in a variety of field projects, especially in surveys in which the study of female specimens is necessary. However, due to the activity of this lure on other noctuids, for comparative surveys, well-founded taxonomic knowledge and working capacity are required.

In situations where selective catches are a requirement and the sampling of males is sufficient, traps baited with Z7-14Ac can successfully be used for *C. vaccinii*.

## Data Availability

The datasets generated during and/or analysed during the current study are available from the corresponding author on reasonable request.
